# Association between the Dietary Inflammatory Index and Risk for Cancer Recurrence and Mortality among Patients with Breast Cancer

**DOI:** 10.3390/nu10081095

**Published:** 2018-08-15

**Authors:** Hyeonjeong Jang, Min Sung Chung, Shin Sook Kang, Yongsoon Park

**Affiliations:** 1 Department of Food and Nutrition, Hanyang University, 222 Wangsimni-ro, Seongdong-gu, Seoul 04763, Korea; jhj0528jhj@hanyang.ac.kr; 2 Department of Surgery, College of Medicine, Hanyang University, 222-1 Wangsimni-ro, Seongdong-gu, Seoul 04763, Korea; 3 Department of Dietetics and Nutrition Service, Asan Medical Center, 88, Olympic-ro 43-gil, Songpa-gu, Seoul 05505, Korea; sskang@amc.seoul.kr

**Keywords:** inflammation, diet, breast cancer, mortality

## Abstract

The dietary inflammatory index (DII) has been associated with breast cancer incidence and survival. However, the association between DII and cancer recurrence and mortality among patients with breast cancer has not been investigated. Therefore, the present study aimed to investigate whether DII was positively associated with risk for cancer recurrence and overall mortality among patients with breast cancer. Among 511 women (51.9 ± 10.7 years; stage 0–3) who underwent breast cancer surgery, 88 had cancer recurrence, and 44 died during follow–up until 213 months (average disease free survival of 84.3 ± 42.4 months and overall survival of 69.3 ± 38.9 months). The DII assessed after surgery (5.4 ± 5.2 months after diagnosis) was significantly higher in patients with recurrence than those without recurrence, and Cox proportional hazards regression analysis showed that it was positively associated with the risk for cancer recurrence (hazard ratio (HR) 2.347, confidence interval (CI) 1.17–4.71) and overall mortality (HR 3.049, CI 1.08–8.83) after adjusting for confounding factors. Disease-free survival and overall survival rates were significantly lower in patients with higher DII scores. In addition, the DII was positively associated with the risk for cancer recurrence according to prognostic factors, such as age (<50 years), premenopausal status, body mass index (≥25 kg/m^2^), HR+, tumor size (>2 cm), and presence of lymph node metastasis. The present study showed that anti-inflammatory diets may decrease the risk of cancer recurrence and overall mortality in patients with breast cancer, particularly those with prognostic factors, such as younger age, premenopausal status, obesity, HR+ breast cancer, tumor size >2 cm, and presence of lymph node metastasis.

## 1. Introduction

Breast cancer is one of the most common cancers in women, and the incidence rate of breast cancer has been increasing worldwide, including in Korea [1,2]. The risk of breast cancer recurrence has been associated with prognostic factors, such as tumor size, lymph node metastasis, American Joint Committee on Cancer (AJCC) stage, histologic grade, and hormone receptor status [3].

There is emerging evidence on the role of inflammatory cytokines on the recurrence of breast cancer [4,5] and mortality [4]. In particular, they modulate hormonal factors associated with breast cancer. Previous studies have reported that inflammatory cytokines were negatively associated with the intake of fiber, carotenoids, and unsaturated fatty acids, but positively associated with the intake of carbohydrates, saturated fatty acids (SFA), and trans-fatty acids [6,7]. The risk for cancer recurrence and mortality was also negatively associated with the intake of fiber, β-carotene, and vitamin C, but positively associated with the intake of fat, SFAs, and unsaturated fatty acids in patients with breast cancer [8,9,10]. In addition, inflammatory cytokines were negatively associated with healthy dietary patterns, but positively associated with western dietary patterns [11,12]. A healthy dietary pattern was also negatively associated with the recurrence of breast cancer and overall mortality [13,14], especially in postmenopausal, hormone receptor-negatively women [15]. However, limited evidence of a relation between an unhealthy dietary pattern and the cancer risk or mortality [14,15].

The dietary inflammatory index (DII), a new tool for assessing the inflammatory potential of the diet, has been associated with inflammatory cytokines, including interleukin (IL)-1β, IL-4, IL-6, IL-10, C-reactive protein (CRP), and tumor necrosis factor-α (TNF-α) [16]. Previous studies have reported that DII was positively associated with the incidence of breast cancer in Chinese, Italian, American, and Swedish women [17,18,19,20], as well as breast cancer mortality in American women [21]. However, no study has investigated the association between DII and cancer recurrence and mortality in patients with breast cancer. Therefore, the present study aimed to investigate the association between DII and the risk for cancer recurrence and overall mortality among patients with breast cancer in Korea.

## 2. Materials and Methods

### 2.1. Patient Population

In total, 530 female patients who underwent breast cancer surgery at the breast cancer clinic of Hanyang University Seoul Hospital were recruited consecutively from June 2000 to August 2017. Some patients were excluded because of the following reasons: implausible energy intake (<500 kcal/day or >4000 kcal/day; *n* = 1), no medical data (*n* = 1), diet survey after cancer recurrence (*n* = 8), and AJCC stage 4 (*n* = 9). Thus, 511 patients were included in the study and followed-up until 213 months with average disease free survival of 84.3 ± 42.4 months and overall survival of 69.3 ± 38.9 months. Recurrence includes a local (*n* = 12) or regional cancer recurrence (*n* = 16), development of contralateral breast cancer (*n* = 10), and distant metastasis (*n* = 50). Moreover, 44 deaths were identified from the medical records during follow-up. Local recurrence was defined as recurrence in residual breast tissue or breast wall, and regional recurrence was defined as recurrence in the ipsilateral regional lymph nodes.

The study was conducted in accordance with the Declaration of Helsinki, and all procedures were approved by the institutional review board of Hanyang University Hospital (HYU 2010-02-001-031) and Hanyang University (HYI-15-070). Prior to enrollment, written informed consent was obtained from all patients.

### 2.2. Data Collection

Information about age; height; body weight; menopausal status; status of estrogen receptor (ER), progesterone receptor (PR), and human epidermal growth factor receptor 2 (HER2); histologic grade; tumor size; lymph node metastasis status; and type of treatment (chemotherapy, hormonal therapy, and radiotherapy) was obtained from the medical records and histopathology reports. Tumor stage and lymph node stage were calculated according to the eighth edition of the AJCC staging system [15]. Body mass index (BMI) was calculated as body weight (kg) divided by height in meters squared (m^2^). Patients were categorized as obese if they had a BMI ≥ 25 kg/m^2^, which is in accordance with the World Health Organization guidelines for Asians [22].

To investigate whether the effect of the DII score was homogeneous across the strata of prognostic factors, we carried out stratified analyses according to age group (<50 and ≥50 years), menopausal status (pre- and postmenopause), BMI (<25 and ≥25 kg/m^2^), hormone receptor status (HR+ and HR−), tumor size (≤2 and >2 cm), and lymph node metastasis status (absence and presence).

A trained dietitian assessed the patient’s diet via a face-to-face interview using a structured 24-h recall questionnaire after breast cancer surgery (5.4 ± 5.2 months after diagnosis). Dietary intake was analyzed using CAN-pro version 4.0 (computer-aided nutritional analysis program; Korean Nutrition Society, Seoul, Korea).

### 2.3. DII

The calculation of the DII was conducted based on a method previously reported by Shivappa et al. [16]. Dietary data were linked to the regionally representative world database that provided a mean and a standard deviation (SD) for each parameter. These parameters were used to identify an individual’s exposure relative to the standard global mean as a z-score. The problem with the right skewing of z-scores was solved by converting them to percentiles. They were then centered on zero, which indicated a null effect on inflammation, by multiplying by 2 and subtracting 1. Finally, the resulting value was multiplied by the corresponding food parameter effect score and summed across these food parameter-specific DII scores to obtain the overall DII score.

A lower DII score indicated a higher consumption of anti-inflammatory foods, whereas a higher DII score showed a higher consumption of pro-inflammatory foods. The DII score that was calculated based on the 24-h recall survey in this study includes data on 34 of the 45 possible food parameters comprising the DII: carbohydrate, protein, total fat, fiber, cholesterol, SFA, monounsaturated fatty acid (MUFA), polyunsaturated fatty acid (PUFA), *n*-3 PUFA, *n*-6 PUFA, thiamin, riboflavin, niacin, vitamin B_6_, vitamin B_12_, β-carotene, vitamin A, vitamin C, vitamin D, vitamin E, folic acid, iron, magnesium, zinc, selenium, pepper, onion, garlic, ginger, turmeric, alcohol, caffeine, and green tea. The remaining 11 missing food parameters were anthocyanidins, flavan-3-ols, flavonols, flavanones, flavones, isoflavones, trans fat, eugenol, saffron, thyme, and rosemary. Because we adjusted for energy intake in the analysis, we did not use this parameter in the calculation of the DII score.

### 2.4. Statistical Analyses

The Statistical Package for the Social Sciences software version 24.0 (SPSS, Inc., Chicago, IL, USA) was used for statistical analyses, and *p*-values < 0.05 were considered statistically significant. The DII scores were categorized into tertiles based on the distribution of all the patients, and the lowest tertile of the DII score was used in the reference group in the analyses. Continuous variables were presented as means ± SD using the independent *t*-test, and the proportions of nominal variables were compared using a chi-square test. To assess for confounding factors, a linear regression analysis was performed between the potential covariates and recurrence status. Moreover, age, BMI, menopausal status, breast cancer subtype, histologic grade, tumor size, lymph node metastasis, AJCC stage, treatment, and energy intake were selected. The daily nutrient and food intakes were compared using the analysis of covariance (ANCOVA) test between the tertiles of the DII score after adjusting for confounding factors. A significant difference was observed in terms of the dietary intake between the groups, and this was presented as different letters using the ANCOVA with Bonferroni post hoc test. The follow-up period began at the date of study entry and ended at the time of the first confirmed cancer recurrence or death (March 2018), whichever came first. The censoring time for the patients without an event was defined as the end date of follow-up. The Kaplan–Meier method was used to calculate cumulative survival probabilities, and the difference between the survival curves was assessed using the log-rank test. The hazard ratios (HRs) and 95% confidence intervals (CIs) of the association between the events and tertiles of the DII score were calculated after adjusting for confounding factors via the Cox proportional hazards regression analysis. *p*-value for trend was calculated using the DII score as a continuous variable in the Cox proportional hazards regression analysis.

## 3. Results

### 3.1. Characteristics of Patients with and without Breast Cancer Recurrence

Patients with cancer recurrence were significantly younger; had a higher proportion of BMI (≥25 kg/m^2^), negative PR expression, histologic grade 3 tumor, tumor that is >2 cm, and stage 3 lymph node metastasis; and underwent chemotherapy, compared with those without cancer recurrence (Table 1).

Forty-one patients with cancer recurrence died as a result of the cancer and three patients without cancer recurrence died as a result of unknown reasons. The DII score of all the patients ranged from −5.87 to +5.48, and the scores were higher in patients with recurrence than those without recurrence. No significant differences were observed between the patients in terms of menopausal status, ER expression, HER2 amplification status, subtype, hormonal therapy, and radiotherapy.

### 3.2. Food and Nutrient Intake of Patients with Breast Cancer According to the DII Score

Patients with the lowest tertile of the DII score significantly consumed more protein, fiber, MUFA, PUFA, *n*-3 PUFA, *n*-6 PUFA, thiamin, riboflavin, niacin, vitamin B_6_, vitamin B_12_, β-carotene, vitamin A, vitamin C, vitamin D, vitamin E, folate, magnesium, selenium, onion, garlic, ginger, and turmeric than those with the highest tertile of the DII score (Table S1). However, no significant differences were observed on the consumptions of carbohydrate, total fat, cholesterol, saturated fatty acid, iron, zinc, pepper, alcohol, caffeine, and green tea between the groups after adjusting for confounding factors.

### 3.3. Association between the DII Score and the Risk for Cancer Recurrence and Overall Mortality in Patients with Breast Cancer

**Figure 1:**
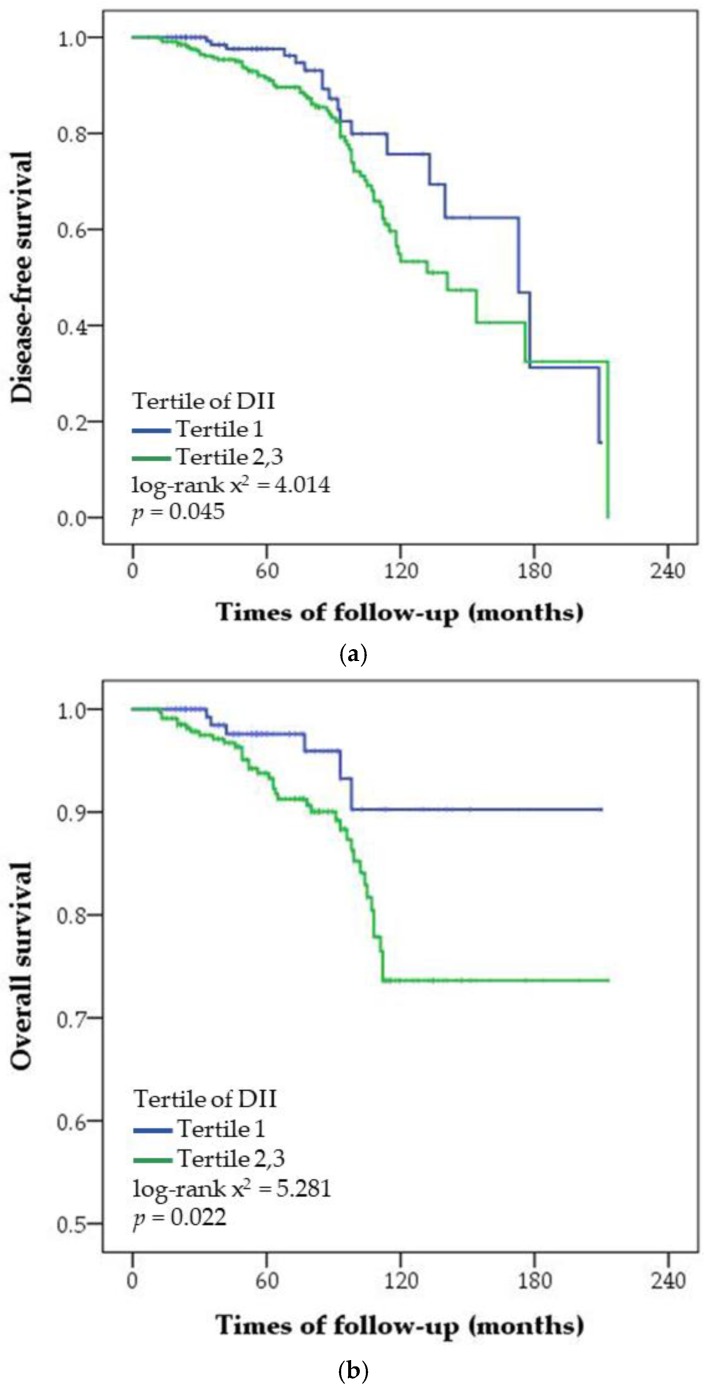
Cumulative disease-free survival (**a**) and overall survival (**b**) according to the tertiles of the dietary inflammatory index (DII) score in patients with breast cancer. Statistical significance was determined using log-rank test and Kaplan–Meier method.

In the multivariable-adjusted regression analysis, DII was positively associated with risk for cancer recurrence and overall mortality after adjusting for confounding factors (Table 2). The risk for cancer recurrence and overall mortality was significantly higher in patients with the highest tertile of the DII score than those with the lowest tertile of the DII score. The patients were followed up for a mean and median period of 69 and 63 (range: 7–213) months, respectively (Figure 1). Because there was no significant difference on cancer recurrence and overall mortality between patients with the middle and highest tertile of the DII score, the middle and highest tertiles of the DII score were grouped and compared with the lowest tertile. Disease-free survival and overall survival in patients with the lowest tertile of the DII score were significantly higher than those with the middle and highest tertiles of the DII score. In the strata of prognostic factors, the risk for cancer recurrence was positively associated with the DII score of patients with breast cancer who are <50 years, premenopausal, and had a BMI ≥25 kg/m^2^, HR+ breast cancer, tumor that is >2 cm, and lymph node metastasis (Table 3). However, the number of deaths was relatively small to confirm the association between DII and risk for mortality according to prognostic factors.

## 4. Discussion

The present study showed that DII was positively associated with the risk for cancer recurrence and overall mortality in patients with breast cancer, and the association was significant in patients aged <50 years, who are premenopausal, and have a BMI ≥25 kg/m^2^, HR+ breast cancer, tumor size >2 cm, and lymph node metastasis.

The association between DII and mortality in patients with breast cancer has not been previously investigated. In addition, in newly diagnosed patients with breast cancer, the association between DII and mortality was controversial [21,23]. DII that was assessed before the diagnosis of breast cancer was positively associated with breast cancer mortality in the Women’s Health Initiative [20], but not with overall mortality in Italian women [23]. The DII score was lower and generally narrower in Italian women than in American women because Italian women adhered more to a Mediterranean diet, which is negatively correlated with DII score [21,23]. In Italian women, the DII score ranged from −2.24 to −0.11, whereas in American women, the DII score ranged from −7.06 to +5.79, which was similar to that of the present study (from −5.87 to +5.48). Moreover, the score was positively associated with inflammatory cytokines, including CRP and IL-6 [24,25], which were also positively associated with the risk for overall mortality in patients with breast cancer. Pro-inflammatory diet has been associated with inflammatory cytokines, particularly IL-6, which is responsible for breast cell migration, invasion, proliferation, and apoptosis [26].

The present study first investigated the positive association between DII and the risk for cancer recurrence in patients with breast cancer. The previous studies showed that DII was positively associated with the incidence of breast cancer in Chinese, Italian, American, and Swedish women [17,18,19,20], but not in American and German women [21,27]. The inconsistencies in the association between DII score and the risk of breast cancer incidence is not fully elucidated. However, it could be attributed to the differences associated with the other underlying characteristics of the population, such as race and menopausal status. The plasma concentration of estradiol and testosterone in premenopausal women was positively associated with the risk of breast cancer [28]. In addition, estrogen replacement therapy increases the risk of breast cancer in postmenopausal women [29]. Previous studies have consistently reported that cytokines, such as TNF-α, IL-1β, and IL-6, increased estrogen synthesis by the activation of aromatase [30,31]. The level of TNF-α was higher in premenopausal than postmenopausal women, and it is positively associated with the risk of breast cancer in premenopausal, but not in postmenopausal women [32]. In postmenopausal women, the association between DII score and the incidence of breast cancer was significant in the Iowa Women’s Health Study [19], but not in the Women’s Health Initiative and Mammary Carcinoma Risk Factor Investigation studies [21,27]. The present study suggested that DII score was positively associated with the recurrence of breast cancer in premenopausal but not in postmenopausal women, which indicates a possible association between DII score and estrogen.

Estrogen is also produced by adipose tissues, thus obesity is a well-known risk factor of breast cancer [33]. In addition, previous studies have consistently reported that BMI was positively associated with breast cancer incidence, recurrence, and mortality in women, particularly in postmenopausal women [34,35]. Morris et al. [36] have suggested that obesity caused subclinical inflammation of adipose tissues characterized by necrotic adipocytes surrounded by macrophages forming crown-like structures, and this is considered a biomarker of an increased risk for breast cancer and poor prognosis. Shivappa et al. [19] have reported that DII score was positively associated with the risk of breast cancer incidence in American women with BMI >30 kg/m^2^, but not in those with a BMI ≤30 kg/m^2^. Consistently, the present study showed that DII score was positively associated with risk of cancer recurrence in women with breast cancer who have a BMI ≥ 25 kg/m^2^, but not in those with a BMI <25 kg/m^2^.

A previous study has reported that DII score was positively associated with risk for breast cancer incidence in Chinese women who are ER+ and PR+, but not in those who are ER+ or PR+ [17]. In the present study, DII score was positively associated with the recurrence of breast cancer in women who are HR+, including those who are ER+ and PR+ and ER+ or PR+, and 80% of women who are HR+ were ER+ and PR+. Patients with ER+ tumors general have a better prognosis than those with ER− tumors. However, ER+ tumors with inflammatory conditions were more likely aggressive, and these tumors were associated with a poor outcome and therapeutic failure [37]. Colleoni et al. [38] have reported that patients with ER+ tumors had a lower recurrence rate within the first five years, but had a higher recurrence rate after five years compared with those with ER− tumors. Therefore, late recurrence may be prevented in patients with breast cancer who have ER+ tumors through dietary intake control.

Breast cancer incidence peaks between the ages of 40 and 50 years in individuals in Asian countries, including Korea, Japan, and China, but between 60 and 70 years in individuals in western countries, such as America, Canada, and Sweden [39]. This variation in incidence may be due to multiple factors, including biological, genetic, ethnic, and environmental factors [39]. Wu et al. [40] have found that high-fat diet (containing 60% calories from fat) increased the levels of IL-6 and TNF-α in young but not old mice, which indicates that the lack of further upregulation of the pre-existing inflammation in response to high-fat diet observed in old mice might be due to age-associated impairment in health conditions that is further deteriorated by high-fat consumption. In Korea, fat intake was 13–19% from calorie in women aged ≥50 years, but 21–27% in women aged <50 years [41]. On the other hand, fat intake of American women was 31–37%, and no difference on fat intake was observed between American women aged ≥50 years and <50 years [42]. In the present study, DII was associated with the risk of cancer recurrence in women aged <50 years who had higher fat intake, but not those aged ≥50 years.

There has been no previous study regarding the association between DII and tumor size or lymph node status, but DII has been shown to associate with inflammatory cytokines such as IL-6 and TNF-α [16]. Previous studies have consistently reported that TNF-α were positively associated with a larger tumor size and presence of lymph node metastasis in patients with breast cancer [5,43]. Tumor size and lymph node status are the major components of the AJCC stage, which is a strong predictor of breast cancer recurrence [3]. The level of IL-6 has been shown to be directly correlated with breast cancer stage, which is indirectly associated with the prognosis of patients [44].

The present study has a few limitations. First, trastuzumab has been used in our patients with HER2-overexpression since July 2010, and this drug improves the survival of patients [45]. In the present study, only 35 out of 511 patients received trastuzumab, which might have a minor impact on the recurrence of cancer and mortality estimation. Second, 11 of 45 food parameters were not available for the DII calculation in the present study. Shivappa et al. [24] have reported that the decrease from 45 to 28 parameters was not a drop off in the predictive capability of the DII, which indicates that the missing food parameters could not have a major impact on the scoring in the present study. Third, dietary intake was only measured once via a 24-h recall, which may not be sufficient in determining the patient’s usual intake. However, women with breast cancer were able to adopt to dietary changes and maintain the changes for up to 24 months [46]. Forth, because of the small number of deaths, we were unable to confirm the association between DII and risk for mortality per prognostic factors. Lastly, although adjustments were made for confounders, unmeasured factors may have affected the results of this study.

## 5. Conclusions

Pro-inflammatory diet may be positively associated with the risk of cancer recurrence and overall mortality in patients with breast cancer, particularly those with prognostic factors, such as younger age, premenopausal status, obesity, HR+, larger tumor size, and presence of lymph node metastasis. However, further studies must be conducted to investigate whether dietary intervention that is focused on inflammation could reduce the risk of cancer recurrence and mortality.

## Figures and Tables

**Table 1:** Baseline characteristics of patients with and without breast cancer recurrence ^1^.

	With Cancer Recurrence (*n* = 88)	Without Cancer Recurrence (*n* = 423)	*p*-Value ^2^
Age (years)	49.57 ± 11.35	52.40 ± 10.50	0.024
Postmenopausal status	38 (43.2)	215 (50.8)	0.192
Body mass index (kg/m^2^)	23.63 ± 3.32	23.03 ± 2.76	0.115
Body mass index (kg/m^2^)			
<25	58 (65.9)	327 (77.3)	0.024
≥25	30 (34.1)	96 (22.7)	
ER expression			
Positive	55 (63.2)	302 (71.7)	0.114
Negative	32 (36.8)	119 (28.3)	
PR expression			
Positive	48 (55.2)	292 (69.4)	0.010
Negative	39 (44.8)	129 (30.6)	
HER2 amplification			
Amplified	25 (28.7)	113 (26.8)	0.718
Not amplified	62 (71.3)	308 (73.2)	
Subtype			
HR+/HER2-	45 (51.7)	256 (60.8)	0.176
HR+/HER2+	18 (20.7)	69 (16.4)	
HR−/HER2+	7 (8.0)	44 (10.5)	
HR−/HER2-	17 (19.5)	52 (12.4)	
Histologic grade			
Grade 1	16 (19.3)	122 (29.9)	0.048
Grade 2	31 (37.3)	160 (39.2)	
Grade 3	36 (43.4)	126 (30.9)	
Tumor size (cm) ^3^			
≤2	31 (36.0)	251 (59.9)	<0.001
>2	55 (64.0)	168 (40.1)	
Lymph node metastasis			
Absence	48 (54.5)	307 (72.6)	0.001
Presence	40 (45.5)	116 (27.4)	
AJCC stage			
Stages 0–2	68 (78.2)	372 (88.6)	0.009
Stage 3	19 (21.8)	48 (11.4)	
Treatment			
Chemotherapy	62 (70.5)	238 (56.3)	0.014
Hormonal therapy	59 (67.0)	318 (75.2)	0.115
Radiotherapy	39 (44.3)	232 (54.8)	0.072
DII score	0.61 ± 1.93	−0.14 ± 2.16	0.003
Death	41 (46.6)	3 (0.7)	<0.001

BMI, body mass index; ER, estrogen receptor; PR, progesterone receptor; HER2, human epidermal growth factor receptor 2; HR, hormone receptor; AJCC, American Joint Committee on Cancer; DII, dietary inflammatory index; ^1^ values are presented as means ± SD or number of patients (percentage distribution) accordingly; ^2^ *p*-values were analyzed using the independent *t*-test for continuous variables and chi-square test for categorical variables; ^3^ largest tumor diameters.

**Table 2:** Cox proportional hazards regression analysis of recurrence and mortality in patients with breast cancer according to the dietary inflammatory index score.

	Tertiles of the DII			*p* for Trend ^1^
	T1 (*n* = 170) −2.37 (−5.87–(−1.07))	T2 (*n* = 170) −0.10 (−1.08–0.97)	T3 (*n* = 171) 2.40 (0.98–5.48)	
**Risk for cancer recurrence**				
No. of patients with/without recurrence	18/152	29/141	41/130	
Adjusted HR (95% CI) ^2^	1.0 (ref.)	1.832 (0.94–3.57)	2.347 (1.17–4.71)	0.019
**Overall mortality**				
No. of deaths/survivors	6/164	17/153	21/150	
Adjusted HR (95% CI) ^2^	1.0 (ref.)	2.403 (0.87–6.65)	3.049 (1.08–8.83)	0.041

Ref., reference; ^1^ estimate of *p* for trend for a linear trend was based on linear scores derived from the medians of the tertiles of DII among all patients; ^2^ adjusted HR (hazard ratio) and 95% CI (confidence interval) were analyzed via Cox proportional hazards regression analysis after adjusting for age, BMI, postmenopausal status, subtype, histologic grade, tumor size, lymph node metastasis, AJCC stage, treatment (chemotherapy, hormonal therapy, and radiotherapy), and energy intake.

**Table 3:** Cox proportional hazards regression analysis of prognostic factors affecting recurrence in patients with breast cancer according to dietary inflammatory index score.

	No. of Patientswith/withoutRecurrence	Tertiles of the DII (HR, 95% CI)^2^			*p* for Trend ^1^
		T1 (*n* = 170) −2.37 (−5.87–(−1.07))	T2 (*n* = 170) −0.10 (−1.08–0.97)	T3 (*n* = 171) 2.40 (0.98–5.48)	
**Age (years)**					
<50	48/189	1.0 (ref.)	3.541 (1.22–10.25)	4.718 (1.63–13.64)	0.006
≥50	40/234	1.0 (ref.)	0.919 (0.35–2.39)	1.636 (0.57–4.74)	0.335
**Menopausal status**					
Premenopause	50/208	1.0 (ref.)	2.101 (0.73–6.04)	3.288 (1.25–8.67)	0.014
Postmenopause	38/215	1.0 (ref.)	1.226 (0.48–3.11)	1.281 (0.41–3.99)	0.669
**BMI (kg/m^2^)**					
<25	58/327	1.0 (ref.)	1.621 (0.73–3.60)	1.980 (0.87–4.51)	0.109
≥25	30/96	1.0 (ref.)	3.427 (0.66–17.94)	8.460 (1.42–50.24)	0.015
**Hormone receptor status**					
HR+	63/325	1.0 (ref.)	1.966 (0.89–4.34)	2.374 (1.05–5.37)	0.045
HR−	24/96	1.0 (ref.)	2.189 (0.54–8.91)	4.260 (0.91–19.87)	0.064
**Tumor size (cm)**					
≤2	31/251	1.0 (ref.)	1.053 (0.38–2.91)	0.843 (0.27–2.61)	0.743
>2	55/168	1.0 (ref.)	1.839 (0.74–4.55)	3.603 (1.47–8.86)	0.004
**Lymph node metastasis**					
Absence	48/307	1.0 (ref.)	0.895 (0.37–2.16)	0.983 (0.38–2.56)	0.998
Presence	40/116	1.0 (ref.)	4.245 (1.37–13.19)	5.287 (1.69–16.54)	0.006

Ref., reference; BMI, body mass index; ER, estrogen receptor; PR, progesterone receptor; HER2, human epidermal growth factor receptor 2; HR, hormone receptor; HR+, ER+, and/or PR+; HR−, ER-, and PR−; ^1^ estimate of *p* for trend for a linear trend was based on linear scores derived from the medians of the tertiles of the DII score among all patients corresponding to prognostic factors; ^2^HR (hazard ratio) and 95% CI (confidence interval) were analyzed via Cox proportional regression analysis after adjusting for age, BMI, postmenopausal status, subtype, histologic grade, tumor size, lymph node metastasis, AJCC stage, treatment (chemotherapy, hormonal therapy, and radiotherapy), and energy intake accordingly.

## Supplementary Materials

The following materials are available online at http://www.mdpi.com/2072-6643/10/8/1095/s1. Table S1: Nutrient and food intake of patients with breast cancer according to dietary inflammatory index score.
