# Supplementary material for: Association between the Dietary Inflammatory Index and Risk for Cancer Recurrence and Mortality among Patients with Breast Cancer
Source: Nutrients. 2018 Aug 15;10(8):1095. doi: 10.3390/nu10081095 (PMC6115987; doi:10.3390/nu10081095)
Supplement: Supplementary file 1 [file nutrients-10-01095-s001.pdf]

Table S1. Daily intake of nutrients and foods in breast cancer patients according to dietary inflammatory index (DII) score [1]

	Tertile of DII			
	**T1 (n = 170)** **−2.37 (−5.87–(−1.07))**	**T2 (n = 170)** **−0.10 (−1.08–0.97)**	**T3 (n = 171)** **2.40 (0.98–5.48)**	***p*-Value [2]**
Carbohydrate (g)	261.30 ± 60.61	221.06 ± 52.97	194.11 ± 53.91	0.612
Protein (g)	70.59 ± 24.24 [a3]	53.52 ± 15.54 [b]	46.11 ± 15.05 [b]	<0.001
Total fat (g)	37.44 ± 15.04	31.69 ± 13.93	27.26 ± 14.30	0.358
Fiber (g)	26.85 ± 7.31 [a]	20.55 ± 4.78 [b]	14.90 ± 4.39 [c]	<0.001
Cholesterol (mg)	262.59 ± 166.91	209.05 ± 159.44	179.43 ± 144.87	0.510
SFA (g)	8.99 ± 9.30	7.05 ± 8.88	6.06 ± 8.80	0.321
MUFA (g)	12.23 ± 12.58 [a]	9.65 ± 12.12 [ab]	7.25 ± 11.37 [b]	0.043
PUFA (g)	8.68 ± 4.98 [a]	6.78 ± 5.09 [b]	4.71 ± 4.73 [c]	<0.001
n-3 PUFA (g)	1.70 ± 1.90 [a]	0.87 ± 1.21 [b]	0.50 ± 0.78 [c]	<0.001
n-6 PUFA (g)	6.16 ± 3.50 [a]	5.13 ± 3.45 [a]	3.41 ± 2.73 [b]	<0.001
Thiamin (mg)	1.31 ± 0.43 [a]	1.05 ± 0.36 [b]	0.86 ± 0.37 [c]	<0.001
Riboflavin (mg)	1.30 ± 0.43 [a]	0.96 ± 0.30 [b]	0.73 ± 0.28 [c]	<0.001
Niacin (mg NE)	16.51 ± 5.03 [a]	12.45 ± 4.17 [b]	9.46 ± 3.58 [c]	<0.001
Vitamin B_6 (mg)	1.93 ± 0.60 [a]	1.46 ± 0.47 [b]	1.09 ± 0.46 [c]	<0.001
Vitamin B_{12} (μg)	9.95 ± 7.77 [a]	6.55 ± 5.81 [b]	5.22 ± 4.73 [b]	<0.001
β-carotene (μg)	6737.98 ± 3962.47 [a]	4068.73 ± 2387.14 [b]	2313.51 ± 1543.28 [c]	<0.001
Vitamin A (μg RE)	1252.73 ± 665.09 [a]	767.70 ± 396.56 [b]	465.54 ± 301.55 [c]	<0.001
Vitamin C (mg)	205.22 ± 131.07 [a]	134.42 ± 92.65 [b]	71.10 ± 45.06 [c]	<0.001
Vitamin D (μg)	5.76 ± 7.61 [a]	3.01 ± 5.16 [b]	1.92 ± 2.61 [b]	<0.001
Vitamin E (mg α-TE)	16.47 ± 4.94 [a]	12.87 ± 4.86 [b]	8.74 ± 4.49 [c]	<0.001
Folate (μg)	656.39 ± 208.11 [a]	456.53 ± 120.81 [b]	326.63 ± 111.14 [c]	<0.001
Iron (mg)	16.99 ± 5.31	16.06 ± 46.28	9.99 ± 4.16	0.519
Magnesium (mg)	113.46 ± 55.42 [a]	74.79 ± 41.96 [b]	41.57 ± 31.33 [c]	<0.001
Zinc (mg)	11.05 ± 4.36	12.27 ± 46.94	6.90 ± 2.58	0.345
Selenium (μg)	88.22 ± 36.74 [a]	72.55 ± 30.01 [a]	56.68 ± 26.39 [b]	<0.001
Pepper (g)	0.05 ± 0.17	0.05 ± 0.17	0.05 ± 0.18	0.685
Onion (g)	26.41 ± 28.64 [a]	16.48 ± 15.80 [b]	11.05 ± 15.73 [b]	<0.001
Garlic (g)	7.77 ± 8.01 [a]	5.17 ± 5.40 [b]	3.33 ± 4.49 [b]	<0.001
Ginger (g)	1.46 ± 4.61 [a]	0.37 ± 1.31 [b]	0.39 ± 2.00 [b]	0.001
Turmeric (g)	0.22 ± 0.84 [a]	0.09 ± 0.51 [ab]	0.02 ± 0.20 [b]	0.017
Alcohol (g)	0.00 ± 0.00	0.21 ± 2.76	0.24 ± 1.97	0.472
Caffeine (mg)	34.70 ± 51.53	40.49 ± 53.48	39.14 ± 52.43	0.778
Green tea (g)	0.04 ± 0.27	0.06 ± 0.42	0.01 ± 0.11	0.395

SFA, saturated fatty acid; MUFA, monounsaturated fatty acid, PUFA, polyunsaturated fatty acid; [1] values are means ± standard deviation (SD); [2] p-values were analyzed using analysis of covariance (ANCOVA) after adjusting for age, body mass index (BMI), postmenopausal status, subtype, histologic grade, tumor size, lymph node metastasis, American Joint Committee on Cancer (AJCC) stage, treatment (chemotherapy, hormonal therapy, radiotherapy) and energy intake; [3] values with different letters within row are significantly different at $p < 0.05$ by ANCOVA with Bonferroni post hoc test.
